# Open Reduction and Internal Fixation Versus Closed Reduction and Maxillomandibular Fixation of Condylar Fractures of the Mandible: A Prospective Study

**DOI:** 10.7759/cureus.21186

**Published:** 2022-01-12

**Authors:** Rathod Prakash, Ramesh K, Aditya M Alwala, Rachana Porika, Manjusha ., Saideep Katkuri

**Affiliations:** 1 Department of Oral and Maxillofacial Surgery, Manthena Narayana Raju (MNR) Dental College and Hospital, Sangareddy, IND; 2 Dentistry, Manthena Narayana Raju (MNR) Polyclinic, Sangareddy, IND

**Keywords:** open reduction, malocclusion, mandible, closed reduction, condylar fracture

## Abstract

Background

The choice of surgical versus nonsurgical treatment for fractures of the condylar process has its own limitations and remains a controversial issue. Improved knowledge of anatomy, technique, and technology combined with adequate experience with careful planning of surgical technique can avoid all the possible complications.

Aim

To compare open reduction and internal fixation with closed reduction and maxillomandibular fixation in the management of condylar fractures.

Materials and method

A prospective study was carried out among 22 patients who had minimally displaced or displaced condylar fractures. The patients were divided into two groups of 11 each: group A patients treated with open reduction and rigid internal fixation and group B patients treated with closed reduction and maxillomandibular fixation. Follow-up examinations were performed at one week, one month, three months, and six months postoperatively.

Results

Preauricular pain was significantly decreased (p < 0.001) in both groups postoperatively but more significantly decreased in the open reduction group. There was a significant improvement in the mouth opening at every follow-up to a maximum mean of 37.36 mm in group A and a mean of 33.64 mm in group B. Significantly more improvement in protrusive and lateral movements and reduced deviation on mouth opening at every follow up was observed in the open reduction group.

Conclusion

Both the treatment options for condylar fractures of the mandible yielded acceptable results with significant clinical differences in terms of occlusion, mouth opening, functional movements, and pain among patients with open reduction.

## Introduction

The face contains many structures that are a predominant cause of morbidity during facial injuries. Maxillofacial (MF) injuries are one of the foremost health problems that remain a serious clinical problem [1]. Every year, over one million people die and more than 25 million are injured from road traffic injuries. Condylar fracture accounts for 11%-16% of all facial fractures and 30%-40% of all mandibular fractures [2] and deserves special consideration apart from the rest of the mandible due to their anatomical differences and their healing potential [3]. There can be a few aspects of maxillofacial trauma management that generate more controversy than the fracture involving the condylar process of the mandible [4].

The treatment goal of condylar fracture should be pain-free mandibular motion, good occlusion, and symmetry [5]. Condylar fractures were traditionally treated with conservative approaches. The choice of treatment method, i.e., conservative or surgical management in adults, is a controversial issue among oral and maxillofacial surgeons around the world. In children, because of a high remodeling capacity of the temporomandibular joint (TMJ), most surgeons prefer the nonsurgical method. This capacity is significant especially during the younger years and decreases while the child is growing. While reaching adulthood, the remodeling capacity has almost vanished [6].

Condylar fracture injuries are frequently underestimated, and the clinical result, especially when conservative therapy is used, might be unsatisfactory. Also, there is reduced interincisal opening, deviation of the mandible, impaired mastication, ankylosis, and internal derangement. Consequently, the pendulum has swung toward accurate anatomical reduction in the hope that it will improve the outcome. The present study aims to compare the outcome of open reduction and internal fixation versus closed reduction and maxillomandibular fixation of condylar fracture of the mandible.

## Materials and methods

The present prospective study was approved by the institutional ethics committee of MNR Dental College and Hospital (D159802051) and conducted in the Department of Oral and Maxillofacial Surgery. The research followed the Helsinki Declaration. Training and calibration of the examiner were carried out in the Department of Oral and Maxillofacial Surgery. Eligible patients included those who are between 15 and 50 years old and who had minimally displaced condylar fracture with one or more of the following presentations: loss of occlusion, shortening of the ramus height associated with molar premature contact, unilateral or bilateral condylar fractures in dentulous or edentulous patients, unilateral or bilateral condylar fractures associated with other fractures, invasion by a foreign body, gross comminuted fracture of the condyle, dislocation of the fracture condyle into middle cranial fossa, and medial/lateral dislocation of the condyle. Patients who had given written informed consent and who were medically fit for surgery under general anesthesia and/or local anesthesia were only included in the study. Subjects with pan facial trauma and who were severely medically compromised were excluded.

A total of 22 patients were included and were divided into two groups of 11 each: group A patients treated with open reduction and rigid internal fixation and group B patients treated with closed reduction and maxillomandibular fixation. A complete history of all the patients was taken preoperatively in a standard case history format. For open reduction and rigid internal fixation, all the patients were operated on under general anesthesia with nasotracheal intubation. A retromandibular approach was used, wherein an incision was given at 0.5 cm below the lobe of the ear of approximately 3-3.5 cm in length and not extending below the angle of the mandible. The skin flap was raised superficial to the parotid fascia and retracted anteriorly. A vertical incision (3-4 cm) was made in the parotid fascia, and the gland was visualized. Blunt dissection through the substance of the parotid gland was done and retracted for better visibility and access. Under direct vision, maxillomandibular fixation was done with 2-mm titanium mini-plates. Monocortical screws (6 mm) were used to fix the plate to the fractured bony fragments.

The closed reduction and maxillomandibular fixation was done with Erich arch bar fixation under local anesthesia. At the time of Erich arch bar fixation, intermaxillary fixation was done with 26-gauge wire. At the first postoperative week, intermaxillary fixation wire was removed, and occlusion was evaluated; then, intermaxillary fixation is done with elastics. Elastics were changed every week postoperatively and replaced with new elastics. All patients were given postoperative instruction and antibiotics, analgesics, and antacid medication prescribed for five days. Following the surgery, the patients were examined at intervals of one month, three months, and six months.

Statistical analysis

The entire results were tabulated and statistically analyzed using SPSS version 18. Comparison of continuous variables among the two groups was done using the ANOVA test, and comparison of categorical variables was done using the chi-square test. An intragroup analysis is possible only for the visual analog scale (VAS) score. Other categorical variables, such as mouth opening, protrusive movement, and lateral excursion, were compared using an independent sample t-test, and occlusion, deviation on mouth opening, and facial nerve function were compared using Fisher's exact test. A p-value of <0.05 was considered statistically significant.

## Results

The mean age of patients who underwent open reduction (group A) and closed reduction (group B) was 31.36 and 26.64, respectively. Group A constituted 81.80% males and 18.20% females, and group B constituted 72.70% males and 27.30% females. In group A, nine patients had subcondylar fractures (81.8%) and two patients had low-level condylar neck fracture (18.2%). With respect to the displacement of fractures, eight patients had medially displaced (72.70%), two patients had laterally displaced (18.20%), and one patient had anteriorly displaced (9.10%) condylar fracture. In group B, five patients had a condylar head (45.4%) and three patients had high condylar neck (27.2%) and subcondylar fracture (27.2%), respectively. Around two patients had medially displaced (18.20%), three patients had laterally displaced (27.30%), one patient had anteriorly displaced (9.10%), and five patients had minimally displaced fractures (45.50%).

The preoperative VAS scores for pain ranged from 4 to 8, with a mean of 6.18 in group A and 5.82 in group B in all 11 patients. The follow-up VAS scores for pain ranged from 4 to 6, with a mean of 4.91 on the first postoperative day among all 11 patients of both groups. In the first week, the mean VAS score reduced to 1.27 in group A and to 2.0 in group B, and most of the patients had no pain at one month, three months, and six months postoperatively. Although the pain significantly decreased (p < 0.001) in both groups postoperatively, the pain was more significantly decreased in the open reduction group than in the closed reduction group (Table 1).

**Table 1:** Pain by Visual Analog Scale

Pain		Mean	SD	P-Value	Post Hoc Test
Group A	Preoperative	6.18	1.40	<0.001	Pre>1w>1m
	1 Day	4.91	1.04		
	1 Week	1.27	1.35		
	1 Month	0.00	0.00		
	3 Months	0.00	0.00		
	6 Months	0.00	0.00		
Group B	Preoperative	5.82	1.40	<0.001	Pre>1w>1m
	1 Day	4.91	1.04		
	1 Week	2.00	1.26		
	1 Month	0.36	0.81		
	3 Months	0.00	0.00		
	6 Months	0.00	0.00		

The preoperative maximum mouth opening ranged from 10 to 21 mm, with a mean of 16.64 in group A patients, whereas in group B, it ranged from 17 to 25 mm, with a mean of 20.18. There was a significant improvement in the mouth opening at every follow-up, to a maximum mean of 37.36 mm in group A and a mean of 33.64 mm in group B. However, mouth opening was more significantly increased in the open reduction group than in the closed reduction group (p = 0.001) (Table 2).

**Table 2:** Maximum Mouth Opening Mouth opening measured in millimeter (mm)

Mouth Opening	Group A (Open)		Group B (Closed)		P-Value
	Mean	SD	Mean	SD	
Preoperative	16.64	2.73	20.18	2.36	0.004
1 Week	24.91	1.97	23.09	0.94	0.012
1 Month	33.00	2.61	28.73	2.80	0.001
3 Months	35.36	1.91	31.55	2.73	0.001
6 Months	37.36	2.16	33.64	2.06	0.001

In comparison with respect to malocclusion, preoperatively, all 11 patients had deranged occlusion in both groups. By the first week, only two patients in group A and four patients in group B had deranged occlusion. All patients had normal occlusion after one month postoperatively in group A (open reduction). However, in one patient in group B (closed reduction), accurate occlusion could not be achieved even at three-month follow-up (Table 3).

**Table 3:** Malocclusion

Occlusion		Group				P-Value
		Group A (Open)		Group B (Closed)		
		N	%	N	%	
Preoperative	No Occlusal Disturbance	0	0	0	0	0.012
	Deranged Occlusion	11	100	11	100	
1 Week	No Occlusal Disturbance	9	81.9	7	63.7	0.008
	Deranged Occlusion	2	18.1	4	36.3	
1 Month	No Occlusal Disturbance	11	100	9	81.9	0.035
	Deranged Occlusion	0	0	2	18.1	
3 Months	No Occlusal Disturbance	11	100	10	90.9	0.035
	Deranged Occlusion	0	0	1	9.1	
6 Months	No Occlusal Disturbance	11	100	11	100	0.035
	Deranged Occlusion	0	0	0	0	

With respect to protrusive movements, preoperatively, all patients had protrusive movements of less than 8 mm, with a mean of 4.09 mm in group A and of 3.45 mm in group B. There was a significant improvement in protrusive movements at every follow-up, to a maximum mean of 8 mm in group A and mean of 6.91 mm in group B. However, when both groups were compared, significantly more improvement in protrusive movements at every follow-up was observed in the open reduction group than in the closed reduction group (Table 4).

Preoperatively, the mean lateral excursion movements on the contralateral side were 4.18 mm in group A and 4.09 mm in group B. The lateral movements were significantly increased in both groups at one month (7.09 and 6.18), three months (7.64 and 6.64), and six months (8.27 and 7.09). When compared between both groups, lateral movements more significantly increased in group A (open reduction) than in group B (closed reduction) (Table 4).

**Table 4:** Protrusive and Lateral Excursion Movements on Contralateral Side Movement on contralateral side measured in millimeter (mm)

	Period	Group A (Open)		Group B (Closed)		P-Value
		Mean	SD	Mean	SD	
Protrusive Movements	Preoperative	4.09	0.54	3.45	0.82	0.044
	1 Week	5.18	0.40	4.36	0.50	<0.001
	1 Month	6.27	0.65	5.73	0.47	0.035
	3 Months	7.18	0.60	6.55	0.69	0.032
	6 Months	8.00	0.63	6.91	0.83	0.002
Lateral Excursion Movements	Preoperative	4.18	0.75	4.09	1.04	0.817
	1 Week	6.00	0.45	5.45	0.82	0.067
	1 Month	7.09	0.70	6.18	1.17	0.039
	3 Months	7.64	0.67	6.64	0.92	0.009
	6 Months	8.27	0.65	7.09	0.83	0.001

Preoperatively, in both groups, all patients had deviation toward the fracture side on mouth opening. By first week, only two patients in group A had deviation, and at one-month, three-month, and six-month follow-up, none of the patients had deviation. In group B, two patients had mouth deviation even at sixth month. When compared between both groups, deviation was more significantly decreased in the open reduction group (group A) than in the closed reduction group (group B) (Table 5).

**Table 5:** Deviation on Mouth Opening

Lateral Movements		Group					
		Group A (Open)		Group B (Closed)		P-Value	
		N	%	N	%		
Preoperative	Deviation	11	100	11	100	>0.99	
	No Deviation	0	0	0	0		
1 Week	Deviation	2	18.1	6	54.6	0.001	
	No Deviation	9	81.9	5	45.4		
1 Month	Deviation	0	0	4	36.4	0.011	
	No Deviation	11	100	7	63.6		
3 Months	Deviation	0	0	3	27.3	0.035	
	No Deviation	11	100	8	72.7		
6 Months	Deviation	0	0	2	18.1	0.09	
	No Deviation	11	100	9	81.9		

Facial nerve function was normal among all the patients of both groups. At first week postoperatively, two patients in group A had transient facial nerve palsy, and at one-month, three-month, and six-month postoperative follow-up, no facial nerve paralysis was seen in the patients. In closed reduction patients, facial nerve paralysis was not seen postoperatively (Table 6).

**Table 6:** Facial Nerve Function

Facial Nerve Palsy		Group				P-Value
		Group A (Open)		Group B (Closed)		
		N	%	N	%	
Immediate Postoperatively	Abnormal	2	18.1	0	0	<0.002
	Normal	9	81.9	11	100	
1 Week	Abnormal	2	18.1	0	0	<0.002
	Normal	9	81.9	10	100	
1 Month	Abnormal	0	0	0	0.0	0.001
	Normal	11	100	11	100	
3–6 Months	Abnormal	0	0	0	0	0.001
	Normal	11	100	11	100	

## Discussion

The choice of surgical versus nonsurgical treatment for fractures of the condylar process remains a controversial issue. In the past, condylar fractures have been treated solely by a closed reduction for various reasons, such as complications involving the facial nerve, technical problems, and scar on the face following surgical treatment, and reasonable good results have been achieved with conservative treatment. However, the closed reduction has long-term complications such as deviation of the mandible, malocclusion, and ankylosis [7]. With the present knowledge of various advantages of open reduction and closed reduction, the present study aimed to compare open reduction and internal fixation versus closed reduction and maxillomandibular fixation in the management of condylar fractures.

In the present study, 21 patients had unilateral condylar fractures, and 18 patients had condylar fractures associated with other mandibular fractures (parasymphysis and symphysis). The cases were selected in accordance with the findings of Haug et al. [8], MacArthur et al. [9], and Ellis et al. [10]. Preauricular pain, one of the most common symptoms following most of the procedures, was seen in all 11 patients who underwent open reduction on the first day postoperatively and among six patients at the first week and then gradually decreased with no pain and tenderness at one-month, three-month, and six-month postoperative follow-up. This initial tenderness is due to the retraction of soft tissues and the manipulation of fracture fragments during surgery. Similar findings were observed in the studies conducted by De Riu et al. [11] and Landes et al. [12]. Similar to the findings of Sforza et al. [13], preauricular pain was noticed in nine patients who underwent closed reduction even at third-month follow-up. However, Oezmen et al. [14] in their study observed that none of the patients had pain after six months of follow-up.

In this study, the maximum mouth opening in the open reduction group was significantly increased postoperatively during six months of follow-up (37.36 mm) than preoperatively (16.64 mm), which is in accordance with the study of Gupta et al. [15] and Takenoshita et al. [16]. This might be due to reduced trismus and TMJ pain with the passage of time, patients performing mouth opening exercises, and fracture healing. Likewise, the maximum mouth opening in the closed reduction group was also significantly increased by the sixth month, although comparatively lesser than the open reduction group. These findings are in line with the study done by Hyde et al. [17].

The discrepancy in occlusion was evaluated in all patients by making the patient close mouth passively in centric occlusion. In the present study, very few patients had occlusal discrepancy only for the first week postoperatively. Initial malocclusion may be attributable to concomitant fractures and transient spasms of masticatory muscles. In line with the findings of Undt et al. [7], Hlawitschka et al. [18], and Schneider et al. [19], none of the patients who underwent open reduction had occlusion discrepancy at one month, three months, and six months postoperatively. The condylar process is usually restored to its pre-traumatic position, or near to it, by open reduction and internal fixation, restoring skeletal continuity and reestablishing normal mandibular position.

In the closed reduction group, occlusion was improved in most of the patients at six months follow-up. These findings correlate with the findings of Landes et al. [20]. However, one of the patients had occlusal discrepancy even at the sixth-month follow-up, and these findings are similar to the study by Singh et al. [21], wherein four patients had malocclusion. This might be due to the reduction in the ramus height or to condyle dislocation from the fossa or the improper reduction of fracture fragments resulting in incomplete anatomical reduction.

The protrusive movements in both groups gradually increased significantly, with an average protrusion of 8 mm in the open reduction method and of 6.91 mm in the closed reduction group at six months follow-up. These results were compatible with the findings of Rutges et al. [22]. Similar to the previous studies of Chrcanovic et al. [23] and Schneider et al. [19], in the present study, the protrusive movement also increased more significantly in group A (open reduction) than in group B, and this might be due to TMJ function impairment after closed reduction, thus limiting mobility.

The lateral movements in both groups gradually increased significantly during the six months follow-up. In the open reduction group, the average lateral movement on the contralateral side was 8.27 mm at six months follow-up, which is in line with the study done by Landes et al. [12] and Ellis et al. [10]. The average lateral movement in closed reduction was 7.09 mm at six-month follow-up, which correlated with the findings of Sforza et al. [13]. Furthermore, the lateral movement was significantly less in group B in comparison with group A, which might be due to impaired TMJ function, limiting mobility, and fracture fragments cannot be reduced as early as closed reduction. However, no significant difference between groups was observed by Haug et al. [8].

The deviation on mouth opening to the fracture side decreased significantly in the open reduction group, and none of them had deviation at three-month follow-up. These results are consistent with the finding of Kotrashetti et al. [24] and Newman [25]. In contrast, Landes et al. [12] observed deviation on mouth opening in two patients out of 11 patients at one-year follow-up. Furthermore, in the closed reduction group, two patients had deviation at three months follow up, and these findings are in line with the previous studies by Kotrashetti et al. [24] and Newman [25]. As the lateral pterygoid function is diminished on the injured side, the contralateral lateral pterygoid pulls the condylar head anteriorly more vigorously. This imbalance causes the chin to deflect to the injured side upon mouth opening.

A study by Nasreen et al. [26] suggests that surgical open reduction and internal fixation of the mandibular condylar fracture is better than the nonsurgical closed reduction, which is in accordance with the present study.

Facial nerve function was assessed in terms of forehead wrinkling, eye closure, facial asymmetry while smiling, and mouth blowing. In the present study, facial nerve function was normal, with only two patients who underwent open reduction having loss of forehead wrinkling and recovered within one month. These results were compatible with the findings of Hyde et al. [17].

The current study's limitations include a small sample size and long-term follow-ups using radiographic examinations to analyze bone repair. Future research should focus on the biological, radiological, and histological outcomes of these procedures with long-term follow-ups to have a better understanding.

## Conclusions

Within the limitations of the present study, it is concluded that both treatment options for condylar fractures of the mandible yielded acceptable results with significant clinical differences in terms of occlusion, mouth opening, functional mandibular movements, and temporomandibular joint pain among patients with open reduction. Furthermore, patients treated with open reduction and internal fixation had a greater anatomical reduction of the condylar process on radiographs. Based on the findings of the present study, open reduction should be indicated in cases of displaced and dislocated condylar fractures with shortening of the ramus, occlusal disharmony, and closed reduction indicated in cases of undisplaced condylar fractures without occlusal disharmony.
